# Evidence for Evolutionary and Nonevolutionary Forces Shaping the Distribution of Human Genetic Variants near Transcription Start Sites

**DOI:** 10.1371/journal.pone.0114432

**Published:** 2014-12-04

**Authors:** Giovanni Scala, Ornella Affinito, Gennaro Miele, Antonella Monticelli, Sergio Cocozza

**Affiliations:** 1 Gruppo Interdipartimentale di Bioinformatica e Biologia Computazionale, Università degli Studi di Napoli “Federico II”, Naples, Italy; 2 Dipartimento di Fisica, Università degli Studi di Napoli “Federico II”, Naples, Italy; 3 Istituto Nazionale di Fisica Nucleare, Sezione di Napoli, Naples, Italy; 4 Dipartimento di Medicina Molecolare e Biotecnologie Mediche, Università degli Studi di Napoli “Federico II”, Naples, Italy; 5 Istituto di Endocrinologia ed Oncologia Sperimentale (IEOS), CNR, Naples, Italy; University of Lausanne, Switzerland

## Abstract

The regions surrounding transcription start sites (TSSs) of genes play a critical role in the regulation of gene expression. At the same time, current evidence indicates that these regions are particularly stressed by transcription-related mutagenic phenomena. In this work we performed a genome-wide analysis of the distribution of single nucleotide polymorphisms (SNPs) inside the 10 kb region flanking human TSSs by dividing SNPs into four classes according to their frequency (rare, two intermediate classes, and common). We found that, in this 10 kb region, the distribution of variants depends on their frequency and on their localization relative to the TSS. We found that the distribution of variants is generally different for TSSs located inside or outside of CpG islands. We found a significant relationship between the distribution of rare variants and nucleosome occupancy scores. Furthermore, our analysis suggests that evolutionary (purifying selection) and nonevolutionary (biased gene conversion) forces both play a role in determining the relative SNP frequency around TSSs. Finally, we analyzed the potential pathogenicity of each class of variant using the Combined Annotation Dependent Depletion score. In conclusion, this study provides a novel and detailed view of the distribution of genomic variants around TSSs, providing insight into the forces that instigate and maintain variability in such critical regions.

## Introduction

The transcription start site (TSS) is canonically defined as the first nucleotide of a transcribed DNA sequence where RNA polymerase begins synthesizing the RNA transcript. TSSs are surrounded by *cis*-acting regulatory sequences, including core and proximal promoter elements located within 1 kb of the TSS, as well as distal promoter elements. Most mammalian promoters are enriched for GC-rich regions, also called CpG islands (CGIs) that serve as structural and functional punctuation marks for transcription [1]. The high GC content of sequences at and around the TSSs of genes suggests a functional relevance for GC-rich elements in higher eukaryotes [1]. Moreover, enrichment of GC-rich regions has been implicated in mutational bias, gene conversion bias and structural plasticity associated with transcription [2]–[6]. Indeed, GC-rich mammalian genes exhibit up to 100-fold greater transcription rates than orthologous GC-poor genes [7].

Populations harbor many genetic variations in promoter regions, most of which are single nucleotide polymorphisms (SNPs) [8]. Evolutionary studies show that different types of nucleotide substitution occur with widely varying rates that may reflect biases intrinsic to mutation and repair mechanisms [9]. These mutations are thought to be strand-asymmetric and context-dependent (CpG vs. non-CpG) [10]–[12]. By analyzing the distribution of SNPs along the length of transcripts, Cui et al. found that SNPs were more abundant near TSSs, with abundance decreasing toward the 3' end. This gradient is more evident for highly expressed genes [13]. Furthermore, packaging of DNA into nucleosomes can limit the accessibility of regulatory proteins involved both in transcriptional regulation [14]–[16] and in replication and, more importantly, in DNA repair [17]–[19]. In vivo studies with yeast and mammalian cells revealed that the DNA packaging into nucleosomes might impair the efficient repair of DNA damage [20]–[23], promoting the emergence of novel mutations.

Novel mutations act as both the substrate for evolution and the cause of genetic disease. Under a neutral model, all the new regulatory mutations should have an equal probability of fixation [24]. In general, evolutionary forces, such as natural selection, drive new alleles either towards a loss from the population (purifying selection), or towards an increasing frequency and possible fixation (positive selection). Given the particular genomic landscape that characterizes promoters and given that most TSSs tend to be conserved between mammals [25]–[26], we can imagine that these regions are generally subjected to selection forces, at least of a conservative kind.

The aim of this work was to characterize the evolutionary and nonevolutionary mechanisms acting near TSSs. To pursue this goal, we analyzed how SNPs belonging to different frequency classes (rare, intermediate and common) and, hence, mainly characterized by different ages, are distributed within the 5000 bp flanking each side of a TSS. In particular, we focused on the relationship between nucleotide and nucleosome characteristics and on the landscape of genomic variation. In addition, we explored the role of evolutionary and nonevolutionary forces in shaping the SNP distribution. Finally, we analyzed the potentially deleterious effects that these kinds of variation can have on molecular processes. We found that in the region under consideration the distribution of variants depends on their frequency and on their localization relative to the TSS. Molecular factors (including sequence features such as CGI or non-CGI, transcriptional activity, presence of nucleosomes) and evolutionary and nonevolutionary forces affecting the fate of mutations in the analyzed regions have been investigated. Finally, we show that variants close to TSSs are potentially more deleterious than those that are more distant, especially in CGIs.

## Results

### Distribution of variants around TSSs depends on their frequencies and on the CpG context

To investigate the distribution of genetic variants around human TSSs, we decided to study their frequency in the 10 kb region surrounding each start site. We downloaded TSS genomic coordinates from the University of California, Santa Cruz (UCSC) database. To adopt a conservative approach, we selected only TSSs having a confidence score ≧20. According to this criterion, we obtained a total of 27,487 TSSs.

The CpG context of TSSs was assessed by dividing TSSs according to their location inside a CGI. We retrieved the genomic coordinates of 27,718 unique CGIs and then divided TSSs into two groups: i) CGI-TSSs, defined as TSSs that were inside a CGI (14,561, ∼53% of the selected TSSs); ii) nCGI-TSS, defined as TSSs located outside CGIs (12,926, ∼47% of the selected TSSs). TSSs were, on average, symmetrically located inside CGIs. We found no significant asymmetries in the distributions of the distances from the center of the CGI (D'Agostino skewness test).

We obtained human genetic variant data from the dbSNP version 138 database. To achieve robust and comparable allele frequencies, we selected only bi-allelic single nucleotide variants contained in the 1000 Genomes Project Variant Catalog that had available allelic frequency values. According to these criteria, we obtained 38,430,070 SNPs that reduce to 2,638,995 considering the only ones located in the 10 kb region surrounding the above selected TSSs. These variants are characterized by minor allele frequency (MAF) values ranging from 0.0001 to 0.5 (mean = 0.043, median = 0.0028, std = 0.099). We then divided variants into four classes according to their MAF values. We named these classes: rare, mid1, mid2 and common. Rare variants (∼21% of all selected variants) were defined by a MAF less than or equal to 4.59×10^−4^. This threshold corresponds to the lowest possible MAF value present in the 1000 genomes Phase 1 release and corresponds to a heterozygous variant being present in only one individual among all 1092 individuals from the Phase 1 release. Common variants (∼32% of the selected variants) were defined according to the canonical criterion of a MAF value greater than 0.01. The remaining variants, with intermediate frequency between rare and common, were partitioned in two groups of equal size (∼23% of all selected variants) and are referred as “mid1” (frequency range: 4.59×10^−4^–0.0014) and “mid2” (frequency range: 0.0014–0.01).

As a first step we compared the mean variant density (SNP/bp) in the 10 kb region around the TSSs versus other genomic regions. As expected, we found that in regions around TSSs mean variant density was lower than in the rest of the genome (0.013 vs. 0.016). Then, for each TSS, we divided the surrounding 10 kb region into 200 bins of 50 bp and we calculated the normalized mean variant frequency for each bin, hereafter denoted by BVF (bin variant frequency) (see Materials and Methods).

**Figure 1:**
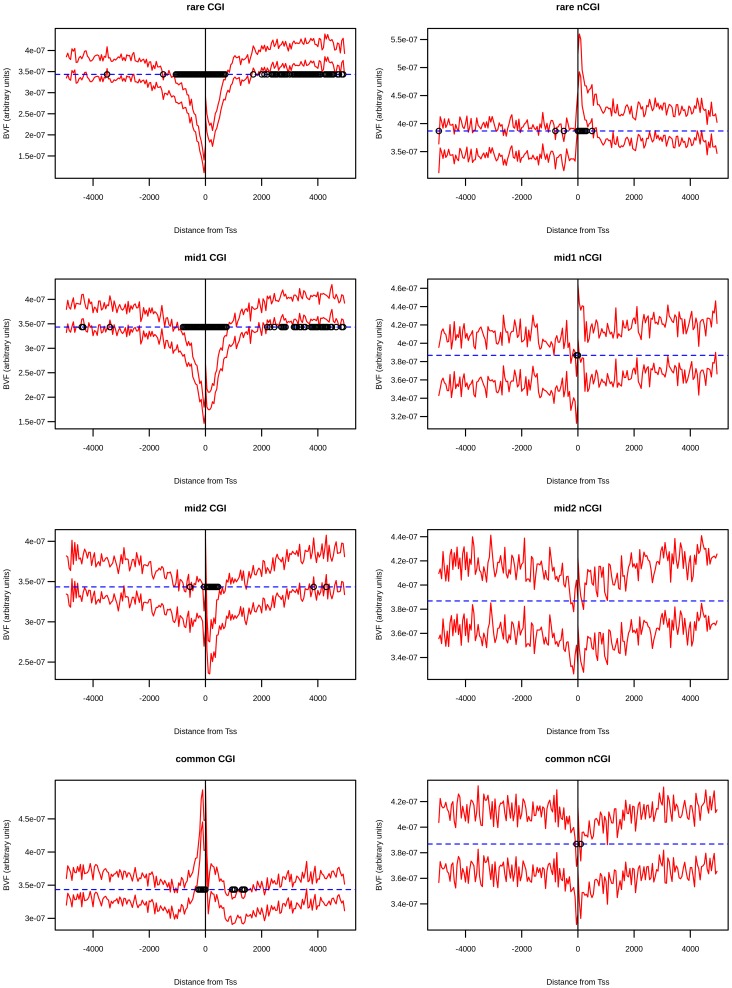
Positional effects of BVF values in the four SNP classes. The two standard error confidence intervals for the observed normalized BVF values (red-dashed lines) are plotted along with its neutral expectation (blue-dashed line) for CGI-TSS frequency classes (left panel) and nCGI-TSS frequency classes (right panel). A dot is placed over the bins whose difference between the observed mean BVF value and the neutral expectation is statistically significant. On the x-axis is the position of the bin relative to the TSS.

Figure 1 shows confidence intervals of BVF values for the four frequency classes for CGI-TSSs and nCGI-TSSs. We observed several peaks and/or depressions in the BVF distribution in several genomic positions. To evaluate if these possible positional effects on BVF values were statistically robust, we compared BVF confidence intervals with a simulated neutral model in which variants were uniformly distributed among different bins and different TSSs (see Materials and Methods).

In the regions around CGI-TSSs we observed a significant positional effect on BVF values for all frequency classes, with a significant deviation from the BVF neutral distribution when in close proximity to TSSs. In addition, for rare and mid1 classes only, we found an extended region of significant deviation between 2000 and 4500 bp downstream of the TSS.

**Figure 2:**
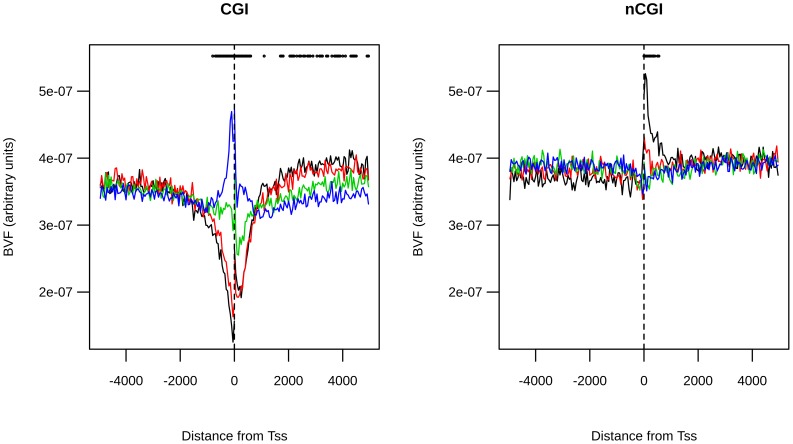
BVF distribution is different among classes. Normalized BVF values for rare (black line), mid1 (red line), mid2 (green line) and common (blue line) variants are reported together on the same plot for CGI-TSSs (left panel) and nCGI-TSSs (right panel). A dot is placed over the bins where the difference of normalized BVF among the four classes is statistically significant. On the x-axis is the position of the bin relative to the TSS.

Looking in more in detail at the rare variant class, for CGI-TSSs we observed a depression of BVF values in the near vicinity of the TSS with a relative peak of BVF values in the first four bins downstream of the TSS. In this restricted region, corresponding to the transcribed 200 bp after the TSS, the BVF values increased ∼1.7 fold in comparison to the corresponding upstream, nontranscribed, region. In regions around nCGI-TSSs we found a significant positional effect only for rare variants. Also, a significant deviation from the BVF neutral distribution was found in the near vicinity of TSSs, but only in the downstream, transcribed region. Figure 1 also shows that BVF signals of the four classes seem to deviate from the neutral model in different manners. This phenomenon is better shown in Figure 2, where all normalized signals are shown on the same graph.

The comparative analysis of normalized BVF signals showed that in some regions they overlap (for example, −5000 to −2000), while in other regions they are very diversely distributed. To investigate this point, we calculated, for each bin, the BVF difference between the two extreme frequency classes (rare and common). We then compared this value (hereafter called BVF-delta) with the corresponding value derived from a neutral model (see Materials and Methods). In Figure 2, dots identify the regions in which the observed BVF-delta value was statistically different from that expected by the neutral model. By this approach, we identified in CGI-TSSs, two regions where the common variant BVF is statistically different from the rare variant BVF. The first region is in the near vicinity of TSSs (with the same notation of Figure 2, we present in Figure S1 a zoom of proximal TSS regions), while the second one is downstream of TSSs (region 2000–4500). It should be noted that, in the first region, the value of the common variant BVF was higher than that of the rare variant, while the opposite relationship was observed in the second region, where the common variant BVF value was lower than that of the rare variant. Figure 2 also shows that in both these regions mid1 and mid2 variants were distributed between rare and common classes, creating a frequency gradient.

In nCGI-TSSs, the common variant BVF was statistically different from the rare variant BVF only in the region immediately downstream of the TSS, where rare variant BVF values were higher than those of the common variant. Also in this case, mid1 and mid2 variant BVF values were distributed between rare and common classes, in such a way as to determine a frequency gradient. To quantify the potential bias introduced by the presence of bi-directional promoters and/or multiple closely located TSSs, we repeated the above analysis excluding regions that host two or more TSSs. By using this conservative filter we retained 58% of CGI-TSSs and 68% of nCGI-TSSs. The results obtained by using such a reduced dataset (see Figure S2) perfectly overlap with those shown for the entire dataset (see Figure 2).

### Distribution of rare variants around TSSs is related to nucleosomal occupancy

**Figure 3:**
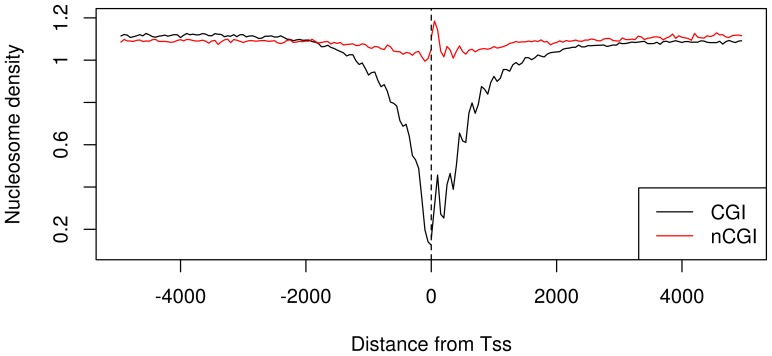
Nucleosome density distribution is different between CGI-TSSs and nCGI-TSSs. The BNP values are plotted together for CGI-TSSs (black line) and nCGI-TSSs (red line). On the x-axis is the position of the bin relative to the TSS.

In general one can expect that variants belonging to different (frequency and/or CGI) classes will be differentially susceptible to the action of different evolutionary forces. It is likely that rare variants are more closely linked to the mutational process and that their frequency is influenced by the presence of mutational “hotspots”. On the other hand, stochastic and evolutionary events (such as drift and selection) can influence the localization of common variants. As a first step, we decided to explore forces potentially affecting the distribution of “rare” variants. It is well known that the presence of DNA packaging structures, for example nucleosomes, can affect the emergence of novel mutations, thus influencing the presence of low frequency variants in a genomic region. Therefore, we decided to search for possible relationships between nucleosome position and rare variant distribution. We downloaded nucleosome positioning scores of the Gm12878 cell line from the UCSC “Stanf Nucleosome” track. By following an analog approach, as for BVF computation (see Materials and Methods), we evaluated the “average nucleosome positioning score” for each bin (BNP), by averaging nucleosome scores for a fixed bin on all TSSs. As expected [27]–[28], nucleosome positioning distribution around the TSS behaved differently for CGI-TSS and nCGI-TSS (Fisher p-value <1e-4), with CGI-TSSs being characterized by a marked depression in nucleosome density in the proximity of the TSS (Figure 3).

**Figure 4:**
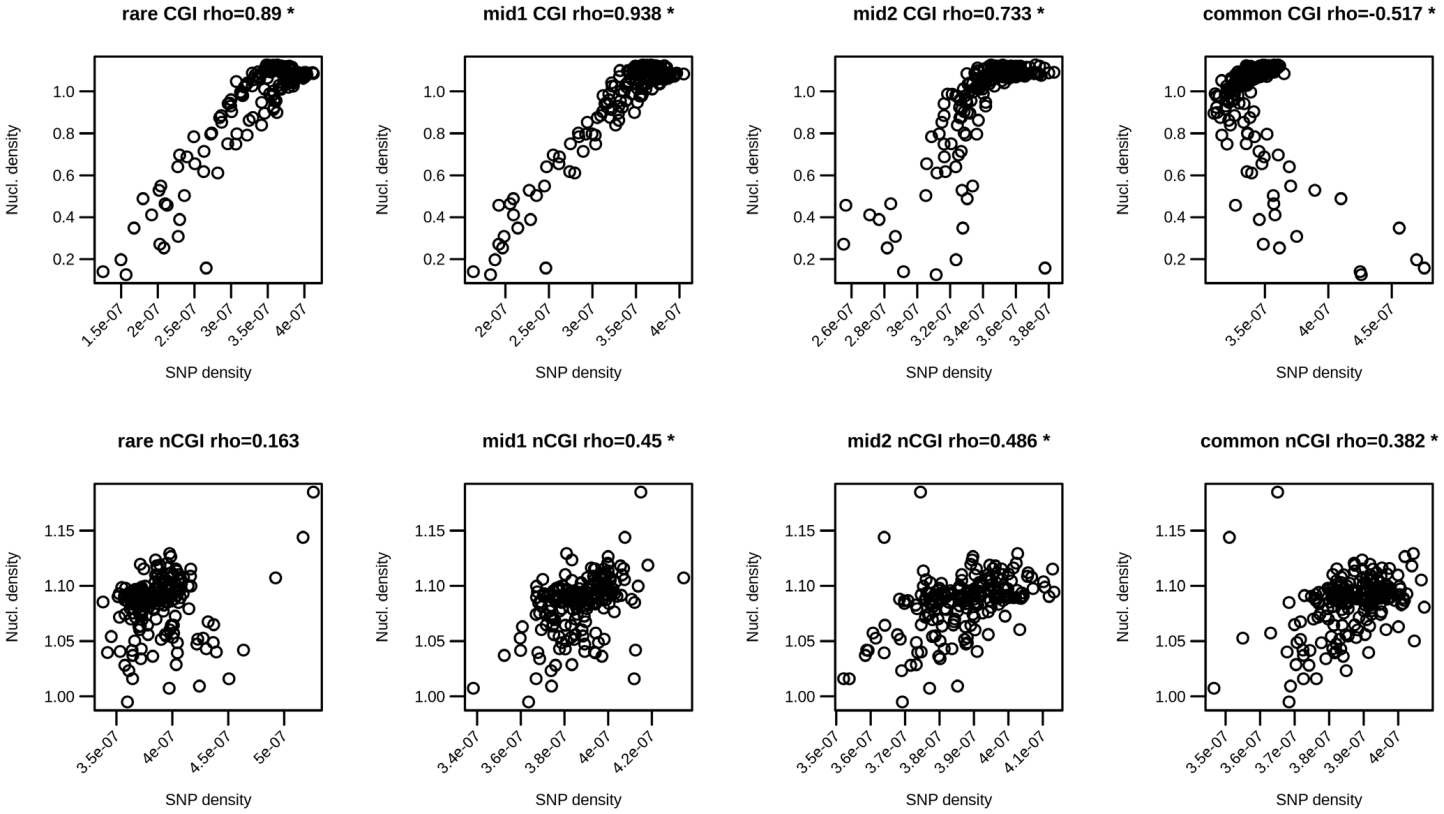
Nucleosome density correlation with SNP density values. Pearson correlations between BNP values and BVF values are reported along with corresponding scatter plots for rare, mid1, mid2 and common variants (from left to right) and for the two TSS classes (CGI-TSSs on the top and nCGI-TSSs on the bottom). * indicates statistically significant correlations.

For each variant frequency class, we plotted the nucleosome positioning score against the variant density in the same bin and for the same TSS class (Figure 4). For all these plots we have then computed the correlation between the two signals. In CGI-TSSs (upper panel), we found a very strong positive correlation for rarer variants that decreases in higher frequency variant classes. In nCGI-TSSs, a weaker correlation was found. The same analysis was conducted for the other available cell line, K562, with similar results (Figure S3).

### Evolutionary and nonevolutionary forces affect the distribution of variants around TSSs

Next we focused on possible forces affecting common variant distribution in the region. In particular, we focused on two forces that may affect allele frequencies within such a genomic area: natural selection and GC-biased gene conversion (gBGC). As is well known, natural selection acts when alleles differ in the resulting fitness of the individual. The effect of natural selection may drive the allele either towards a reduced frequency/loss in the population (purifying selection), or towards an increasing frequency/fixation (positive selection).

gBGC is a recombination-associated process that favors some alleles over others independently of the fitness conferred. gBGC is a process in which GC/AT (strong/weak) heterozygotes are preferentially resolved to the strong allele during gene conversion. We reasoned that this force could be particularly important in CpG rich regions near TSSs.

**Figure 5:**
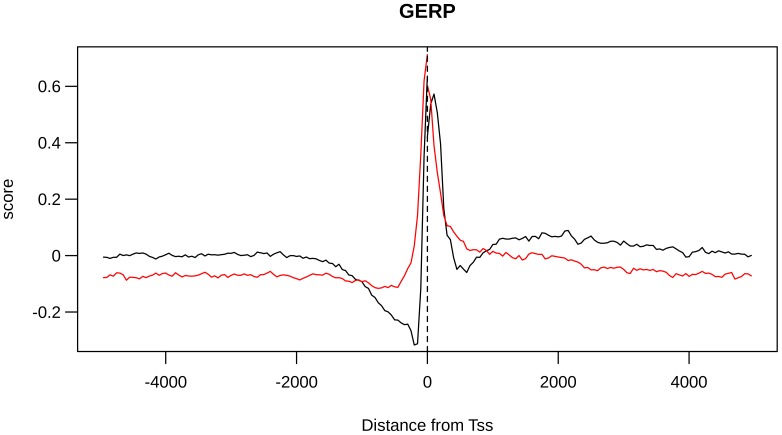
GERP distribution is different between CGI-TSSs and nCGI-TSSs. The BGS values are plotted together for CGI-TSSs (black line) and nCGI-TSSs (red line). On the x-axis is the position of the bin relative to the TSS.

**Figure 6:**
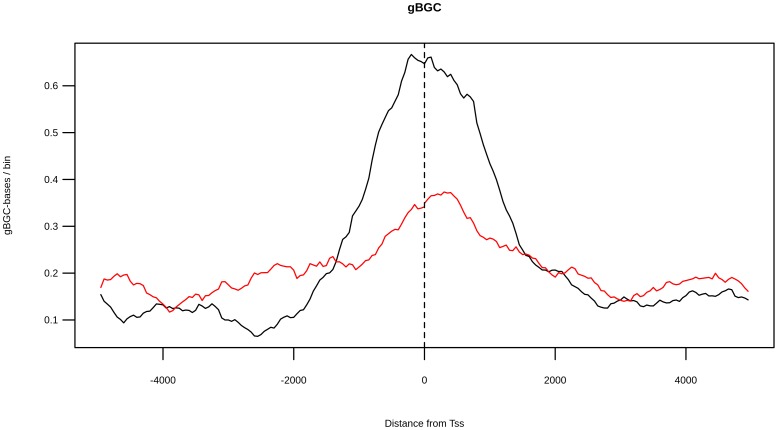
gBGC score distribution is different between CGI-TSSs and nCGI-TSSs. The BBS values are plotted together for CGI-TSSs (black line) and nCGI-TSSs (red line). On the x-axis is the position of the bin relative to the TSS.

**Figure 7:**
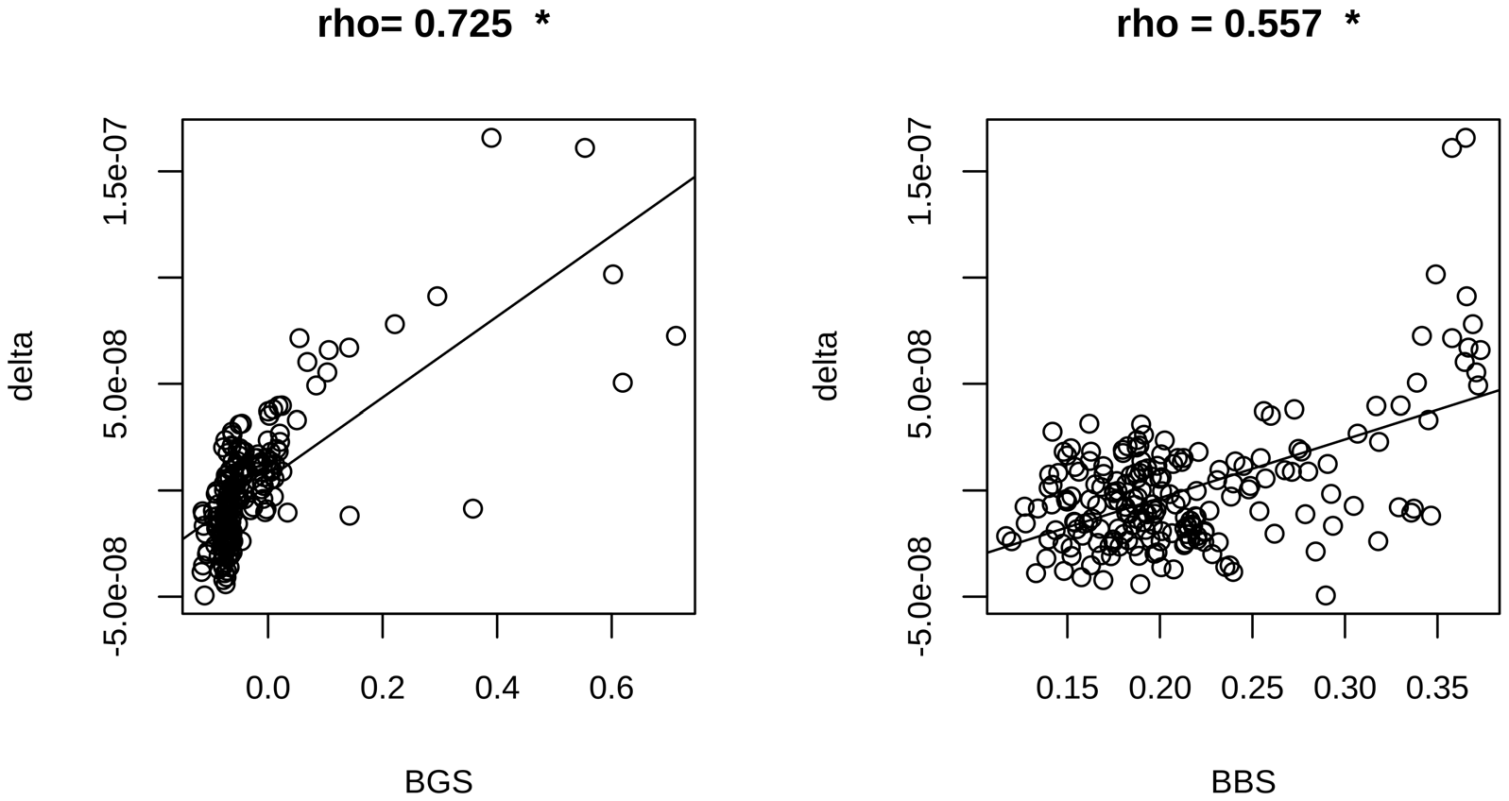
BVF-delta is strongly correlated with GERP in nCGI-TSSs. Pearson correlations between BGS and BVF values (left panel) and between BBS and BVF values (right panel) are reported along with corresponding scatter plots for nCGI-TSSs. * indicates statistically significant correlations.

To identify possible signatures of natural selection, we analyzed the conservation profiles of the analyzed regions by Genomic Evolutionary Rate Profiling (GERP) scores [29]. High values of this score indicate a lower level of substitutions among species (with respect to a neutral value derived by applying a maximum likelihood evolutionary rate estimation), hence indicating a high evolutionary conservation. To evaluate the possible presence of gBGC phenomena, we used “phastBias” gBGC track from UCSC. By using this track we obtained bases predicted to be influenced by GC-biased gene conversion (gBGC bases) [30]. We determined the “bin average GERP score” (BGS) by computing, for a fixed bin, the GERP values averaged over bin loci and over all TSSs (Figure 5). By using an analog process, we obtained the “bin average gBGC score” (BBS) by computing, for a fixed bin, the average number of gBGC bases over all considered TSSs (Figure 6). We found that the BGS distribution was different between CGI-TSSs and nCGI-TSSs (Fisher p-value <10^−4^). For both TSS classes, we observed a peak in the region ∼100 bp upstream and ∼200 bp downstream of the TSS. A region with negative BGS signal was found 200–700 bp upstream of the CGI-TSS only. Also for the BBS signal we found different distributions for CGI-TSSs and nCGI-TSSs, with a peak in the region from ∼2000 bp upstream to 2000 bp downstream of the CGI-TSSs. Then, we plotted BGS, BBS and BVF-delta values for nCGI-TSSs and CGI-TSSs (Figure S4, Figure S5). For nCGI-TSSs, Figure S4 shows an apparent direct correlation among the three variables. We tested these correlations and we found a strong positive correlation between BVF-delta and BGS values (Pearson correlation coefficient = 0.725, *p*-value <2.2 10^−16^) and a weaker correlation (Pearson correlation coefficient 0.557, *p*-value <2.2 10^−16^) between BVF-delta and BBS values (Figure 7). For CGI-TSSs, Figure S5 shows a more complex pattern. In particular, an inverse correlation appeared between BVF-delta and both BGS and BBS in the near vicinity to TSSs, whereas a direct correlation for the same signals was present in the complementary distal regions.

**Figure 8:**
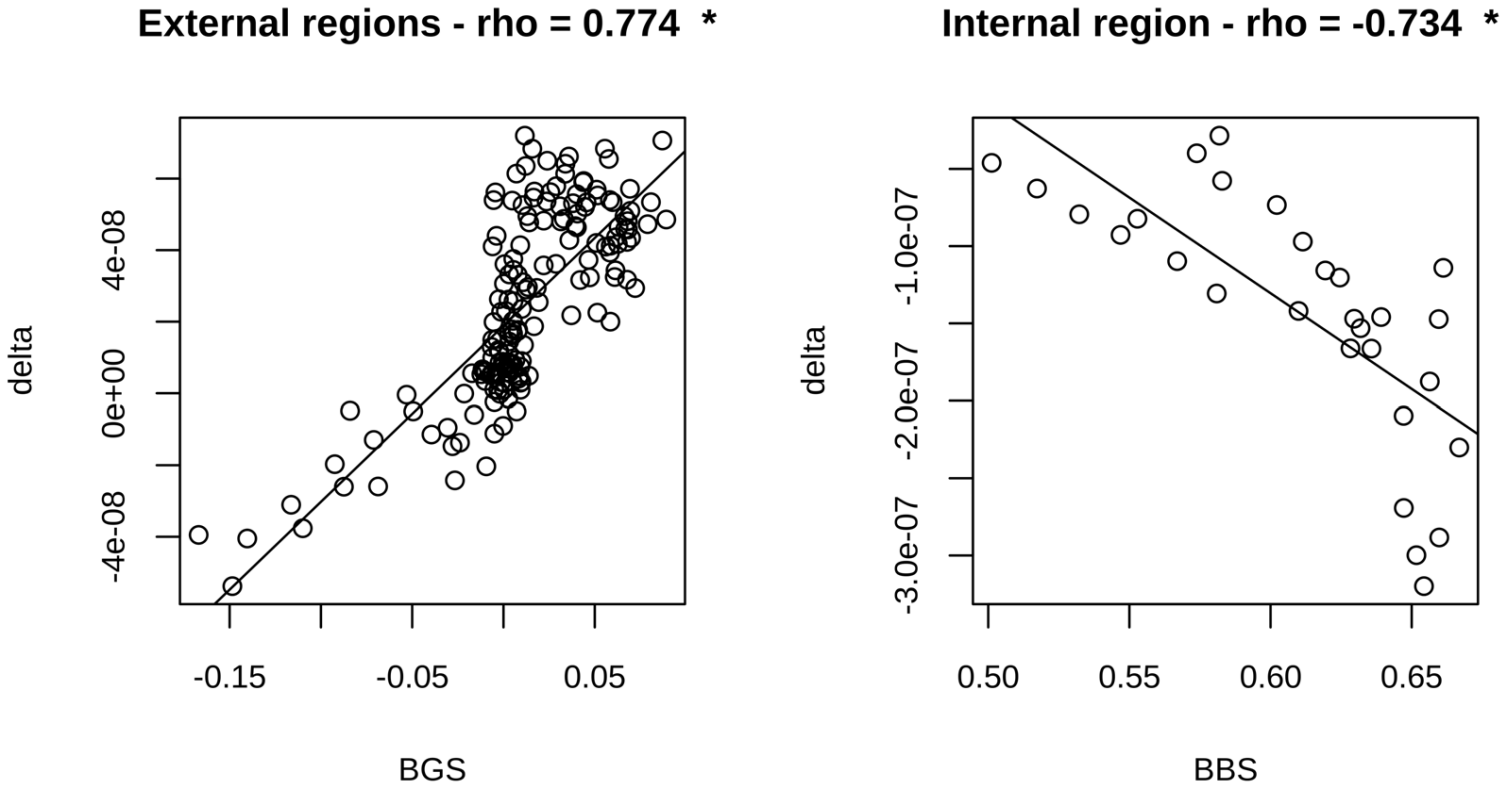
GERP and gBGC correlations for inner and outer regions. Pearson correlations between BGS and BVF values in the outer region (left panel) and between BBS and BVF values in the inner region (right panel) are reported along with corresponding scatter plots for CGI-TSSs. * indicates statistically significant correlations.

To better quantify such an involved pattern, we considered a generic symmetric window around the TSS and evaluated the correlations of BBS vs. BVF-delta and BGS vs. BVF-delta as a function of the window size. Since the first correlation, as an absolute value, was significantly larger than the second one in proximal (inner) regions, we chose to calculate the correlation of BBS and BVF-delta in the inner region and between BGS and BVF-delta in the complementary one. By using a window-based approach (see Materials and Methods), we were able to split the whole region into an inner one (∼700 bp region flanking the TSS), where BVF-delta is mainly correlated with BBS, and an outer complementary one, where BVF-delta is mainly correlated with BGS. Analysis of the two regions showed a strong positive correlation (rho = 0.77, p-value <2.2 10^−16^) between BGS and BVF-delta in the external region (Figure 8, left panel) and, conversely, a strong negative correlation (rho = −0.73, p-value  = 5.78 10^−6^) in the inner region between BVF-delta and BBS (Figure 8, right panel).

### Variants around TSSs could have functional effects

**Figure 9:**
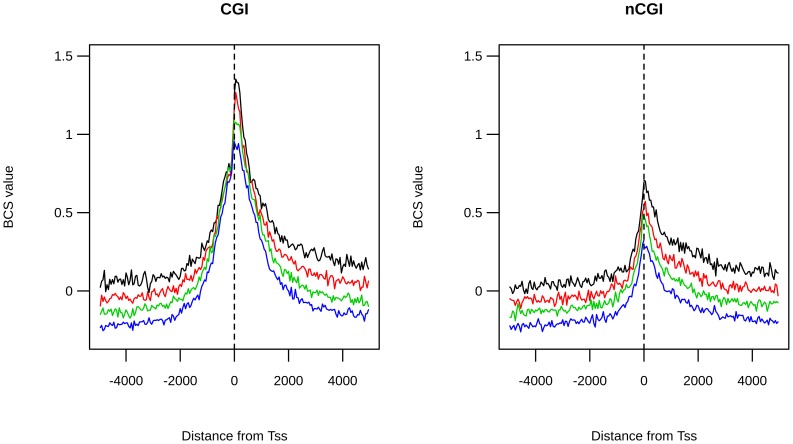
Deleteriousness scores show different position effects among TSS classes. BCS values are plotted on the same region for rare (black line), mid1 (red line), mid2 (green line) and common variants (blue line) for CGI-TSSs (left panel) and nCGI-TSSs (right panel). On the x-axis is the position of the bin relative to the TSS.

As a last step, we analyzed the potential pathogenicity of each class of variants. We did this by analyzing the CADD (Combined Annotation Dependent Depletion) score [31]. High values of this signal characterize variants that are likely to have deleterious effects, namely its *deleteriousness*. For both TSS classes and for each of the variant frequency classes we computed the “bin average CADD score” (BCS) obtained by computing, for a fixed bin, the CADD values averaged over bin variants and over TSSs (Figure 9). As expected, we found a statistically significant difference (see Materials and Methods) among the four signals, with SNP deleteriousness values that generally decreased as the frequency of a variant increases. In all considered classes, deleteriousness increased moving toward the TSS from both sides. Finally, for each frequency class, significantly higher values of deleteriousness were seen for CGI-TSSs compared with nCGI-TSSs in the region proximal (∼1300 bp) to the TSS site (see Materials and Methods).

## Materials and Methods

### TSS selection

Genomic coordinates of human TSSs were downloaded from the UCSC track “switchDbTss”. This track reports data collected in the “switchDB”, an open-access online database of human TSSs from “SwitchGear Genomics” (http://www.switchgeargenomics.com), where the location of 131,780 TSSs throughout the human genome is determined by integrating experimental data. The database provides, for each site in the genome, a score that encodes the confidence to have a TSS placed in that position. To exclude unreliable data, we retained TSSs whose confidence score was greater than or equal to 20; *N_TSS_* = 27,487 (∼21% of the total).

### Variant selection

The human genomic variants used in this study were downloaded from the “snp138” UCSC table. This track consists of all human genomic variants reported in dbSNP build 138: a massive collection of molecular variants (including SNPs, insertions, deletions) coming from heterogeneous studies. We selected only variants from dbSNP that: i) include the term “1000GENOMES” in the “submitters” field; ii) have the value of the field “class” equal to “single”; iii) have the value of the field “alleleFreqCount” equal to 2; iv) have both the two comma separated values of the field “alleleNs” greater than 0; and v) have the sum of the two comma separated values of the field “alleleNs” greater than 1000. By applying this filter, we obtained 2,638,995 variants. Since there is no explicit representation of the MAF in this dataset, we computed it by taking the minimum of the two allele frequencies for each variant. This was computed by using the two comma separated values reported in the dbSNP field “alleleNs”.

### CpG island selection

The genomic coordinates of CGIs were obtained from the UCSC “CpgIslandExt” track. In this track CGIs were predicted by searching the sequence one base at a time, scoring each dinucleotide (+17 for CG and −1 for others) and identifying maximally scoring segments. In this dataset, to define a CGI the following criteria were used: i) to have a GC content of 50% or greater; ii) to have a length greater than 200 bp; and iii) to show a ratio greater than 0.6 of observed number of CG dinucleotides to the expected number, calculated on the basis of the number of Gs and Cs in the segment under analysis. UCSC CGI files also contain data related to sequence for alternative haplotypes (present mainly in chromosome 6, for the inclusion of alternative versions of the MHC region). Of course, in our analysis we filtered the file to exclude these duplicated data.

### Nucleosome positioning scores

Nucleosome localization data were downloaded for two cell lines, namely Gm12878 and K562, from the ENCODE UCSC tracks “wgEncodeSydhNsomeGm12878Sig” and “wgEncodeSydhNsomeK562Sig”, respectively [32]. These tracks contain density signal maps of nucleosome positions produced by the MNase-seq technique. In these tracks, nucleosome-positioning data were generated without immunoprecipitation, therefore no selection was applied for histone modifications such as methylation or acetylation.

### GERP evolutionary scores

To evaluate the impact of conservative evolutionary forces we used GERP. This approach identifies, by multiple alignments and maximum likelihood evolutionary rate estimation, genomic regions with a substitution rate lower than that expected under neutral hypothesis, suggesting that they are under functional constraint. Therefore GERP is able to detect signatures of past purifying selection. Conservation data for the regions of interest were downloaded from the “allHg19RS_BW” UCSC track. This track contains an evolutionary score obtained by GERP. Constraint intensity at each individual alignment position is quantified in terms of a "rejected substitutions" score, defined as the number of substitutions expected under neutrality minus the number of substitutions "observed" at the position [33]. Expected and observed substitutions were computed by alignment of hg19 to 35 other mammalian species.

### gBGC scores

Human genomic regions predicted to be influenced by GC-biased gene conversion were downloaded from the UCSC “phastBias” track. This signal was derived by phastBias prediction model applied to the human-chimp lineages of the phylogenetic tree as described in [30]. This method, predicts gBGC tracts using a four-state hidden Markov model (HMM) (conserved, neutral, conserved with gBGC, neutral with gBGC). In particular, the phastBias track contains only regions for which the assigned posterior probability to be affected by gBGC is greater than 0.5.

### Combined Annotation Dependent Depletion scores

CADD scores were generated using the method of Kircher et al. [31]. These scores are computed through a Support Vector Machine approach that is based on 63 different annotations spanning a wide range of data types. These include conservation metrics such as GERP, phastCons, and phyloP; functional genomic data such as DNase hypersensitivity and transcription factor binding; transcript information such as distance to exon-intron boundaries or expression levels in commonly studied cell lines; and protein-level scores such as Grantham, SIFT, and PolyPhen. In particular these scores can be interpreted as the extent to which the annotation profile for a given variant suggests that the variant has deleterious effects. Scores were pre-computed for all 8.6 billion possible single nucleotide variants of the reference genome, as well as all variants from the 1000 Genomes and ESP variant releases. We downloaded CADD data for all 1000 Genomes variants from http://cadd.gs.washington.edu. The file contains, for each variant, the genomic position and two scores, namely the "raw" and "scaled" C-Scores. For the purposes of this work we employed the raw score.

## Statistical Analysis

### Definition of upstream and downstream regions

Upstream and downstream regions were defined on each strand for each TSS by considering the direction of the transcription process. For each TSS, the upstream region was defined as the 5000 bp on the 5' side if the TSS is located on the coding strand and as the 5000 bp region on the 3' side if the TSS relies on the noncoding strand. Conversely, the downstream region was defined as the 5000 bp on the 3' side of the TSS if this is located on the plus strand and as the 5000 bp on the 5' side if the TSS relies on the minus strand.

### Bin Variant Frequency calculation

Let *TSS(i)* be the position of i-th TSS. *Bin(i,j)* is defined as the genomic interval with extrema

- *TSS(i)*+j*50, *TSS(i)*+(j-1)*50 if j > 0,
- *TSS(i)*+j*50 and *TSS(i)*+(j+1)*50 if j < 0,

with *j* in −100,…,−1,+1,…,+100 and *i* in 1…*N_TSS_*.

Let us denote with *vars(i,j)* the number of variants falling into the *Bin(i,j)* region. We defined the normalized mean variant frequency for the j-th Bin as:
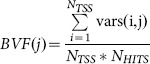
where
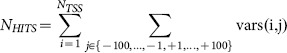



### Positional effects on BFV values

For each considered subset of SNPs, we considered a neutral model in which variants are uniformly distributed among bins and among all TSS regions. In particular we determined the mean of this null distribution as:
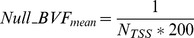



We then tested, for each bin j, the difference between the observed mean values *BVF(j)* and the *Null_BVF_mean_* by means of a two-sided t-test. All 200 *p*-values were finally corrected using the Bonferroni method.

To investigate the presence of different positional effects on *BVF* values among classes, we calculated, for each bin, the difference between rare variant-normalized *BVF* (*BVF_rare_*) and common variant-normalized *BVF* (*BVF_common_*) values:
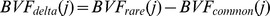



We evaluated the statistical significance of the *BVF_delta_* values by testing their difference from the neutral value 0. The above differences were tested by means of a two-sided t-test and the corresponding *p*-values were finally corrected using the Bonferroni procedure.

### Bin Nucleosome Positioning calculation

Let *NUC(i,j)* be the set of nucleosome density values from *“wgEncodeSydhNsomeGm12878Sig”* or *“wgEncodeSydhNsomeK562Sig”* (for Gm12878 and K562 cell lines, respectively), associated with sites placed inside *Bin(i,j)*, and let 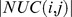 be its cardinality. If we denote with *score(i,j)* the sum of all nucleosome scores from *NUC(i,j)*, we can compute the “average Nucleosome Positioning score” for the j-th Bin as:
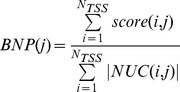



To evaluate the statistical difference of the whole *BNP* signal between CGI-TSSs and nCGI-TSSs we compared the values of each pair of corresponding bins by means of the two-sided t-test, thus obtaining 200 *p*-values. We then tested if the observed local differences suggest a global difference of the whole signals. Since each one of the above-mentioned t-tests can be considered as an independent test of the null hypothesis and CGI-TSSs and nCGI-TSSs are sampling the same signal, we combined the obtained *p*-values by means of Fisher's method: 
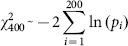



Correlations between BNP vs. BVF-delta were obtained by means of Pearson's product moment correlation coefficient. The statistical significance of each correlation was obtained by evaluating the test statistic: 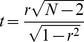
with *r* representing the correlation coefficient and *N* the number of observations (bins). Under the null hypothesis of no correlation between the two tested variables, *t* is distributed as a Student's T with *N-2* degrees of freedom.

### Evolutionary analysis

Let *GER(i,j)* be the set of GERP score values from the “allHg19RS_BW” sites whose genomic position fall inside the Bin(i,j) region, and let 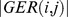 be its cardinality.

By denoting with *score(i,j)* the sum of all scores belonging to *GER(i,j)*, we can compute the “Bin average GERP Score” for the j-th Bin as:
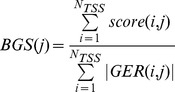



Analogously, let us denote with *bgc(i,j)* the number of genomic positions from the “phastBiasTracts3” UCSC track falling into the *Bin(i,j)* region. We defined the normalized Bin average gBGC Score for the j-th Bin as:
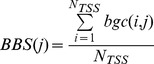



To evaluate the statistical difference of the whole *BBS (BGS)* signal between CGI-TSSs and nCGI-TSSs, we compared the values of each pair of corresponding bins by means of two-sided t-tests, thus obtaining 200 *p*-values. We tested if the observed local differences suggest a global difference of the whole signals using the same procedure described for BNP values.

For CGI-TSSs, we tried to split the whole region into two subregions where the effects of gBGC or BGS are respectively dominating. To this aim we applied the following method. Starting from the TSS site, at each iteration *i* (starting with i = 2 till i = 98) we considered the region defined by bins with an index of [*−i,i*] and the complementary one defined by [−100, −i[U]+i, +100]. At each iteration we evaluated the correlation between BBS and BVF-delta in the region [−i,i] and between BGS and BVF-delta in the region [−100, −i[U]+i, +100]. Let *j* be the index *i* for which the absolute value of the product of the two correlations is maximized, then we chose [−j,+j] as the region under strong BGC influence and the complementary region (with respect to the whole analyzed region) as the one under strong BGS influence (Figure S6). We can safely compute the product of correlations since BGS and BBS signals are supposed to be independent.

Correlations between both BBS vs. BVF-delta and BGS vs. BVF-delta were obtained by means of Pearson's product moment correlation coefficient. The statistical significance of each correlation was obtained by evaluating the test statistic: 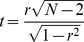
with r representing the correlation coefficient and N the number of observations (bins). Under the null hypothesis of no correlation between the two tested variables t is distributed as a Student's T with N-2 degrees of freedom.

### Deleteriousness analysis

Let *DEL(i,j)* be the set of deleteriousness values (from the CADD 1000 genomes dataset) associated with sites placed inside *Bin(i,j)*, and 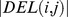 its cardinality. If we denote with *score(i,j)* the sum of all CADD scores belonging to *DEL(i,j)*, we can compute the “bin average CADD score” for the j-th Bin as:
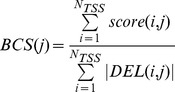



For each bin j, we tested the difference in the mean *BCS* value between the four frequency classes by means of a one-way analysis of variance test, finally correcting *p*-values with the Bonferroni procedure.

For each bin j, we tested the difference in the mean *BCS* value between CGI-TSSs and nCGI-TSSs of the four frequency classes by means of a two-sided t-test and Bonferroni correction.

Upstream versus downstream regions were compared by Wilcoxon rank sum tests.

### Statistical significance assessment

This study was conducted considering a *p*-value of 0.001 as statistically significant. All statistical analyses were performed using R ver. 2.10.1 software.

## Discussion

In this paper, we analyzed the distribution of SNPs inside a 10 kb region flanking human TSSs. We divided SNPs into four classes according to their frequency (rare, two intermediate classes, and common) to explore in detail the genetic variability of the region, and to gain insight into the forces that generate and maintain this variability.

We found that the distribution of variants in these regions depends on their frequency class, and on their localization relative to TSSs. Furthermore, splitting TSSs in CGI-TSSs (located inside a CGI) and nCGI-TSSs (not located inside a CGI), showed that the distribution of variants is generally different for the two subsets. Nevertheless, for both CGI and nCGI-TSSs a peak of rare variants immediately downstream of the TSS was observed. This region corresponds to approximately the first 200 bp of transcribed DNA. In general, rare alleles are younger than others [34], and are hence more closely related to the mutational process. In this sense, the peak of rare alleles that we found next to TSSs could be broadly considered as a signature of an increased mutation rate. Our results show that, proportionally, this peak is higher in CGI (1.7 fold) than in nCGI-TSSs (1.15 fold). This suggests that, owing to their intrinsic structural and functional features, mutagenic phenomena are more active in CGIs-TSSs than in nCGI-TSSs. CGIs not only act as promoters, but are also associated with several other functionally-relevant genomic features, including recombination hotspots [35]–[39], the presence of transposable elements [40], domain organization and nuclear lamina interactions [41], origins of replication [42]–[46], and local mutational processes [47]. Furthermore, transcription-associated mutagenic processes and transcription coupled repair are more active in CGI-TSSs than in nCGI-TSSs. Indeed, CGI-promoters are generally associated with constitutively expressed genes in all cell types (housekeeping genes) [48], whose expression is necessary for the maintenance of cell physiology, while nCGI promoters are generally associated with highly tissue-specific genes and tend to have more restricted expression patterns.

Previous studies reported SNPs arising as a result of transcription-associated mutagenic processes [15], [17], [49] and a strong inhibitory effect of nucleosomes on mutation reparability by limiting the access of repair proteins [50–52]. Since our experiments suggest transcriptional effects on mutation rate in a specific location (about 200 bp downstream the TSS), we explored if these effects could be related to nucleosome occupancy.

Studying the relationship of the rare allele distribution with nucleosome occupancy score, we found a very strong positive correlation for CGI-TSS variants, and a weaker, but still significant, correlation for nCGI-TSS variants. Interestingly, Mu et. al. found a periodical distribution of SNPs around TSSs in regions associated with CGIs [47], and explained such result in terms of location of nucleosomes. In [48], Tolstokurov et al. failed to find a positive correlation between SNPs occurrence and nucleosome occupation in a 1 Kb flanking region of human TSSs. This result can be explained by the fact that the authors did not separate SNPs according their frequency, considering all SNPs together. Our data support the hypothesis that transcription-related mutational phenomena could be related to a reduced efficiency of the repair mechanisms in the regions occupied by nucleosomes [50,53]. Indeed, damage within the nucleosome core is repaired at a rate of about 10% of that for naked DNA [20].

A strong reduction of the rare variants frequency in CGI-TSSs is also shown, but this is a known characteristic of CGIs in which the incidence of mutations is depressed. Indeed, if, on the one hand, transcriptional-related mutagenic processes are more active in CGIs, on the other hand, purifying selection might then counteract the loss of CpGs to preserve the existence of CGIs for regulatory processes [54]. As expected [27], [28], nucleosome distribution around TSSs is different for CGI-TSSs and nCGI-TSSs. In the case of CGI-TSSs, we observed a marked depression in nucleosome density in the 1 kb flanking regions. This is in agreement with the expectation that constitutive genes, such as those associated with CGI-promoters, exhibit a nucleosome-free region at their TSS to provide space for the assembly of the transcription machinery [55].

In contrast, in nCGI-TSSs we observed a peak just downstream of the TSS, corresponding to a high intrinsic nucleosome occupancy. This finding can be explained by the fact that CGI-promoters are associated with housekeeping genes [48], while nCGI promoters generally have more restricted expression patterns, depending, for example, on the developmental stage or cell type. In fact, in the absence of physiological requirements, it could be advantageous to keep nCGI regulatory sites masked with nucleosomes to minimize risks of inappropriate utilization and aberrant transcription.

A common characteristic of both CGI-TSSs and nCGI-TSSs is a neutral regime observed for regions more than 1000 bp upstream of the TSS. This is to be expected because of the significant distance from the TSS and generally being a noncoding region. In contrast, the presence of a gradient in the relative frequencies, with increasing values from common to rare variants, could reliably testify to purifying selection that, in this specific case, would preserve coding regions from the accumulation of deleterious mutations and prevent deleterious mutations from reaching common frequencies. This last observation is also supported by the strong correlation of the BVF-delta signal with GERP scores in regions where rare variants (younger) are more frequent than the common (oldest) ones.

Such features can be observed in the coding regions of both nCGI-TSSs and CGI-TSSs. In particular, for the former, the selection is active in a restricted region in the near downstream vicinity of TSS only. The same kind of selection, in the same region, is also at work for CGI-TSSs as indicated by a strong depletion of common variants with respect to the specular region upstream. Interestingly, the gradient in relative frequencies extends far into the downstream region for CGI-TSSs only. This difference with the nCGI-TSSs could be related to the strong enrichment of housekeeping genes among CGI-TSSs. As described in [56], housekeeping genes evolve more slowly than tissue-specific genes in terms of both coding and core promoter sequences. Selective constraint differences could arise from differences in gene function and expression. As housekeeping genes play a key role in the maintenance of most cells, strong purifying selection acts to preserve their normal function, whereas for tissue-specific genes, which are expressed in few tissues, the impact of a deleterious mutation is less than that of housekeeping genes.

Interestingly, there is the intriguing presence of a sharp peak of common variants in the near vicinity of CGI-TSSs. This peak was completely absent in nCGI-TSSs and implies that forces influencing this pattern could be, at least in part, related to the CpG content of the region. A CpG content-related force, able to influence the distribution of allele frequencies within a population, is the GC-biased gene conversion (gBGC). According to such conversion phenomenon, GC/AT heterozygotes are preferentially resolved to GC/GC homozygotes during gene conversion. This may be seen as a kind of back mutation that tends to restore the intermediate gene frequencies causing a nontrivial stable distribution of gene frequencies at equilibrium [57]. In other words, the effect of back mutation interferes with the possible loss or fixation of variants, with the result of an enrichment of common frequency variants with respect to the case in which only genetic drift is at work (neutral scenario).

We analyzed this hypothesis by looking at the difference in relative frequencies between rare and common variants. Under the neutral hypothesis we expected that this quantity would have a vanishing mean value. Any statistically significant deviation from this value suggests that evolutionary (like selective pressure) and/or nonevolutionary (like gBGC) forces are acting in that specific region.

As observed by Polak and Arndt [35], CGIs contain a mutational signature of GC-biased gene conversion, which determines an enrichment of GC nucleotides in CGIs [58]. Also, according to Galtier and Duret [59] the BGC process is not a mutagenic process that introduces de novo mutations into the genome, but instead it increases the fixation probability of GC alleles over AT alleles [59], [60]. Our results seem to show the simultaneous action of drift, selective pressure and gBGC for CGI-TSSs. In particular, the depletion of common variants with respect to the specular upstream region could be explained by the presence of a purifying selection, whereas the larger relative frequency of common variants with respect to the rare variants testifies the action of gBGC. In this sense, gBGC could compete against, and slow the effects of purifying selection. In some cases, gBGC overcomes purifying selection and leads to the fixation of deleterious AT → GC mutations that would be eliminated in the absence of gBGC [61], [62], thus counteracting natural selection [63]. The limited extension of the region where gBGC phenomena dominate over selection is marked by the inversion point of the gradient. Remarkably, such a point almost corresponds to the downstream end of the CGI.

Finally, when we analyzed the deleteriousness of each class of variants, we found that it decreases as the frequency of variants increases, suggesting that rare variants are more deleterious than common ones. Furthermore, the deleteriousness is higher in CGI-TSSs than in nCGI-TSSs, in accordance with the structural and functional importance of CGIs, as explained above.

## Supporting Information

Figure S1 **BVF distribution is different among classes – zoom of the proximal TSS region.** Same notation of Figure 2.
(TIFF)

Figure S2 **BVF distributions after excluding regions that host two or more TSSs.** Same notation of Figure 2.
(TIFF)

Figure S3 **Correlation of nucleosome density with SNP density for the K562 cell line.** Pearson correlations between BNP and BVF values are reported along with corresponding scatter plots for rare, mid1, mid2 and common variants (from the left to right) and for the two TSS classes (CGI-TSSs top and nCGI-TSSs bottom). * indicates statistically significant correlations.
(TIFF)

Figure S4 **Overlapping normalized values of BGS, BBS and BVF-delta values for nCGI-TSSs.** The z-scores for BBS (green line), BGS (red line) and BVF-delta (black line) are plotted for the same region. On the x-axis is the position of the bin relative to the TSS.
(TIFF)

Figure S5 **Overlapping normalized values of BGS, BBS and BVF-delta values for CGI-TSSs.** The z-scores for BBS (green line), BGS (red line) and BVF-delta (black line) are plotted for the same region. The grey lines delimit the region defined under strong gBGC influence. On the x-axis is the position of the bin relative to the TSS.
(TIFF)

Figure S6 **Regions under strong BBS and/or BGS influence.** Figure shows, for each pair of inner-outer regions defined by the distance reported on the x-axis, the absolute value of the Pearson correlation for BBS and BVF-delta in the inner region (red line), the absolute value of correlation for BGS and BVF-delta in the outer region (black line) and the product of the two correlations (blue-dashed line). Red dots are placed where the correlation between BBS and BVF-delta is statistically significant and black dots are placed where the correlation between BGS and BVF-delta is statistically significant. The vertical dashed line represent the distance for which the value of the product of the two correlations is maximized.
(TIFF)

Data S1 **Instructions for obtaining data.**
(DOCX)
