# Supplementary figures and images for: Evidence for Evolutionary and Nonevolutionary Forces Shaping the Distribution of Human Genetic Variants near Transcription Start Sites
Source: PLoS One. 2014 Dec 4;9(12):e114432. doi: 10.1371/journal.pone.0114432 (PMC4256220; doi:10.1371/journal.pone.0114432)

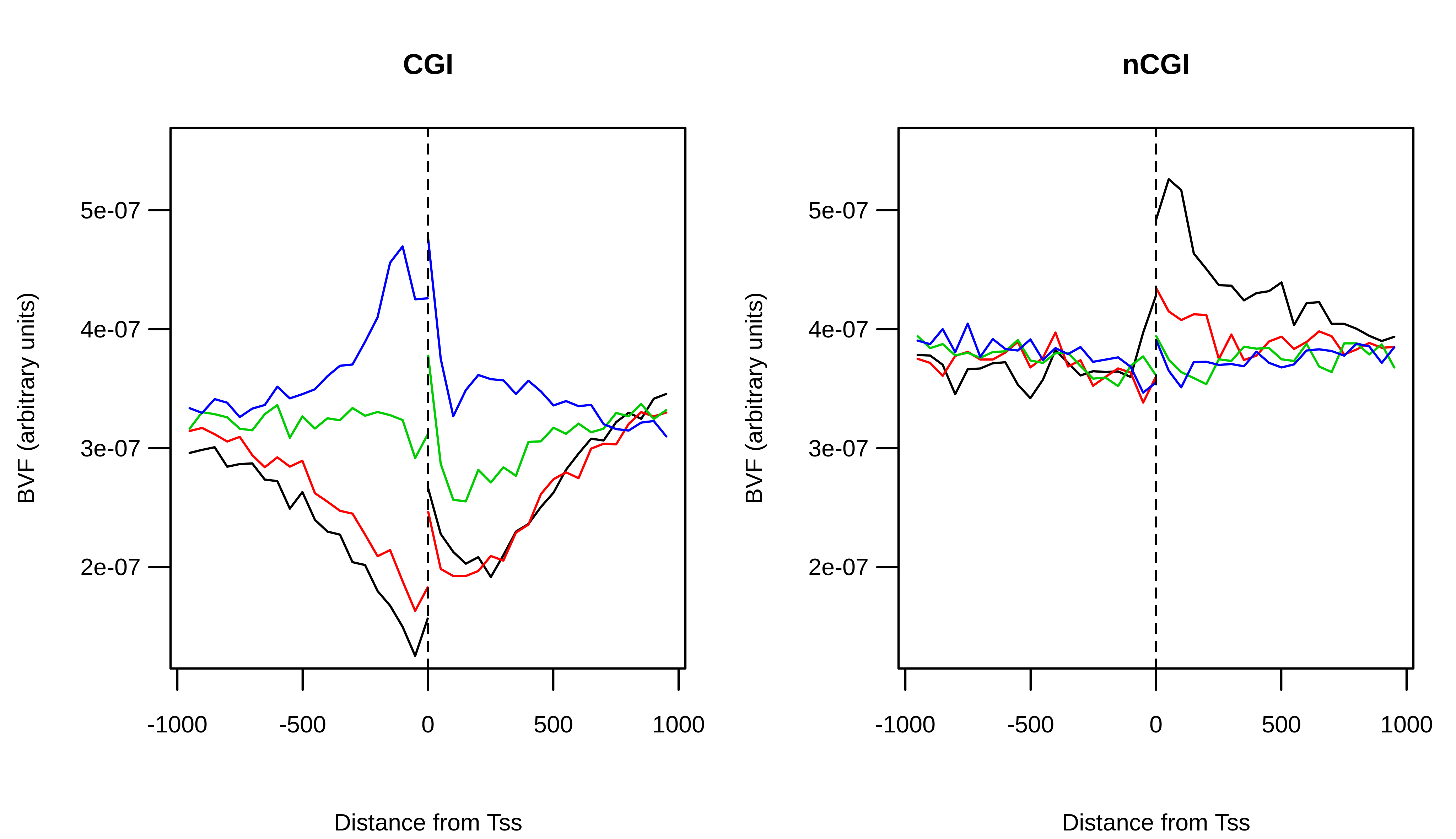

Supplement: Figure S1 — BVF distribution is different among classes – zoom of the proximal TSS region. Same notation of Figure 2. (TIFF) [file pone.0114432.s001.tiff]

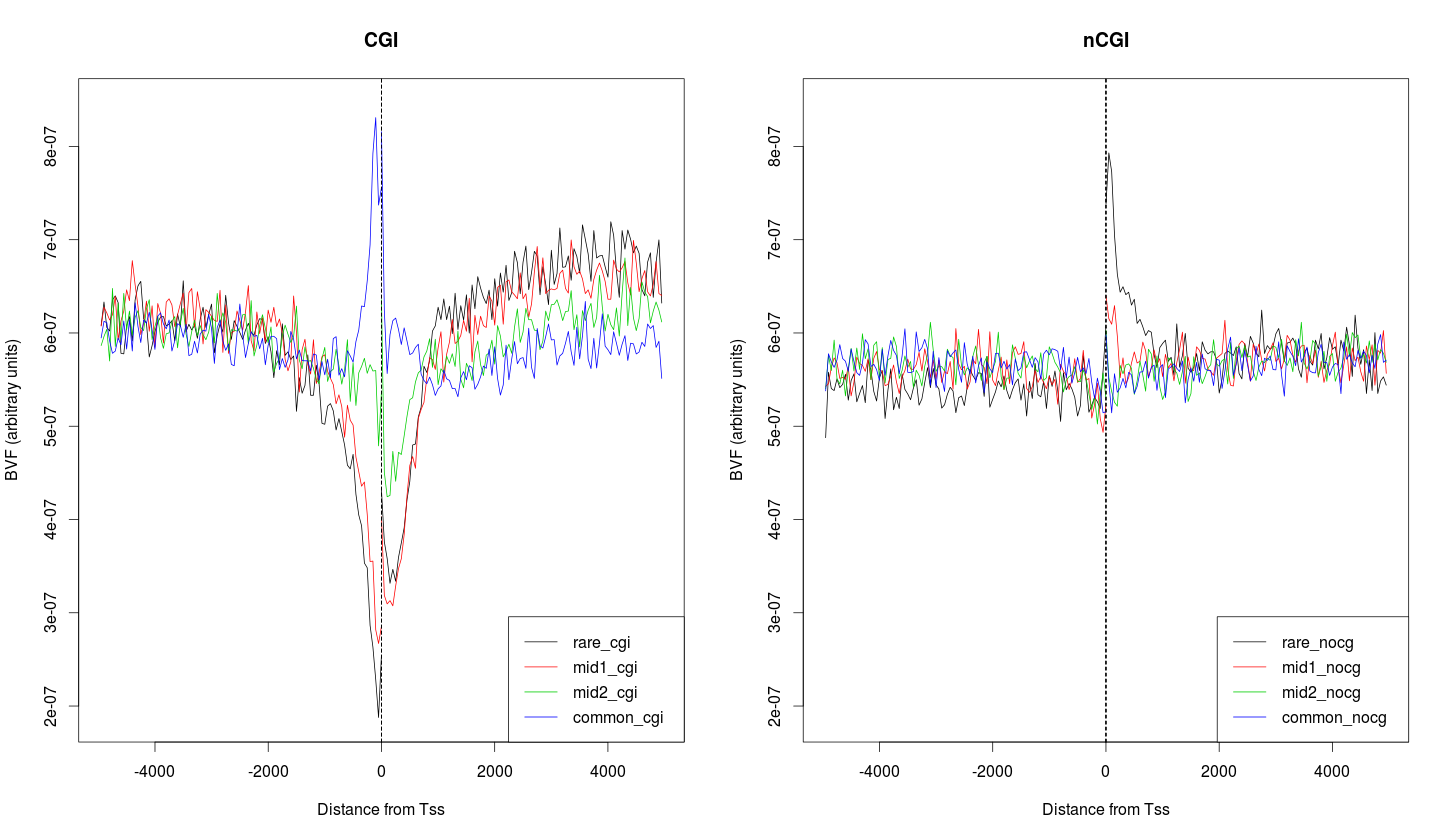

Supplement: Figure S2 — BVF distributions after excluding regions that host two or more TSSs. Same notation of Figure 2. (TIFF) [file pone.0114432.s002.tiff]

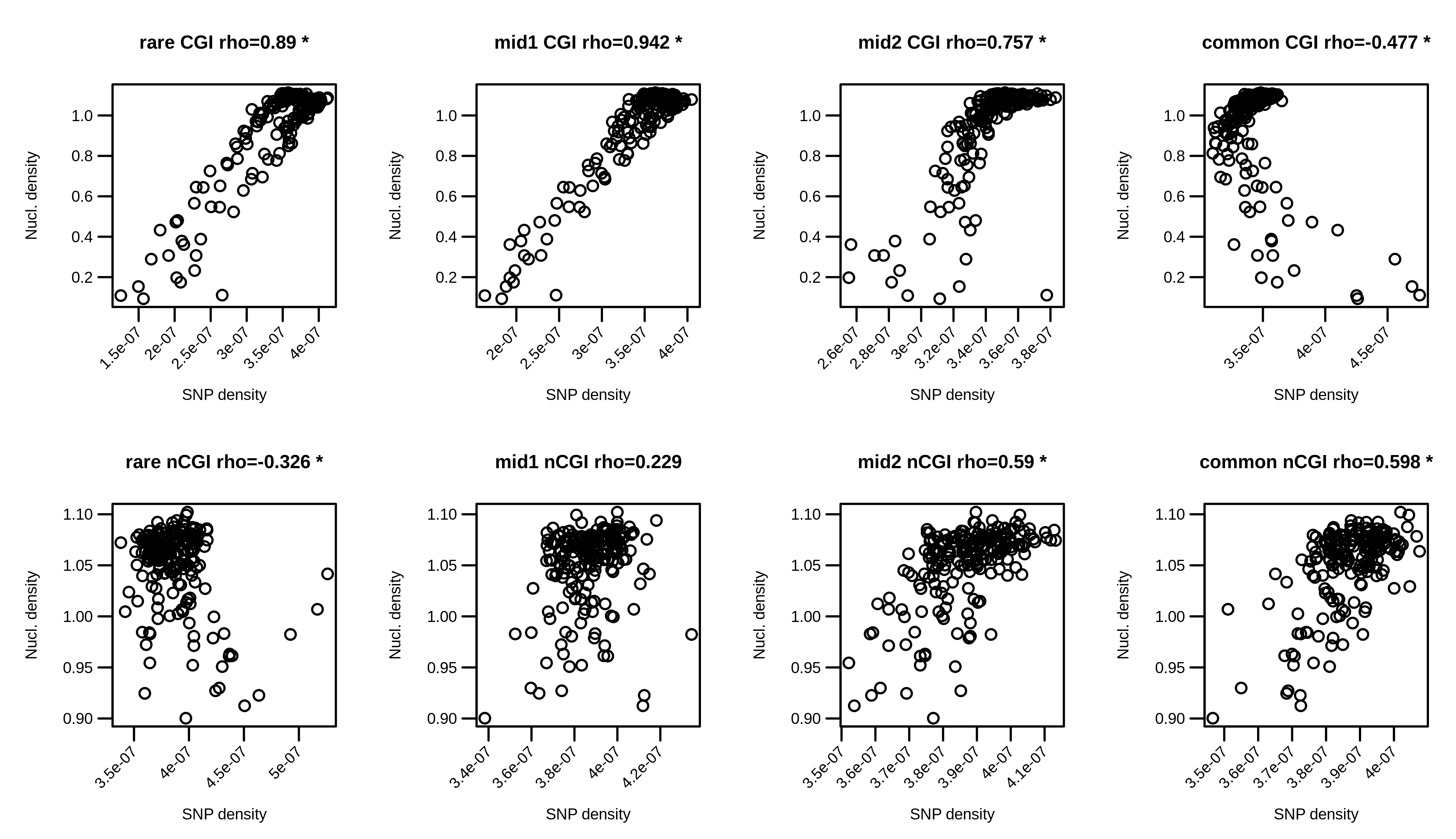

Supplement: Figure S3 — Correlation of nucleosome density with SNP density for the K562 cell line. Pearson correlations between BNP and BVF values are reported along with corresponding scatter plots for rare, mid1, mid2 and common variants (from the left to right) and for the two TSS classes (CGI-TSSs top and nCGI-TSSs bottom). * indicates statistically significant correlations. (TIFF) [file pone.0114432.s003.tiff]

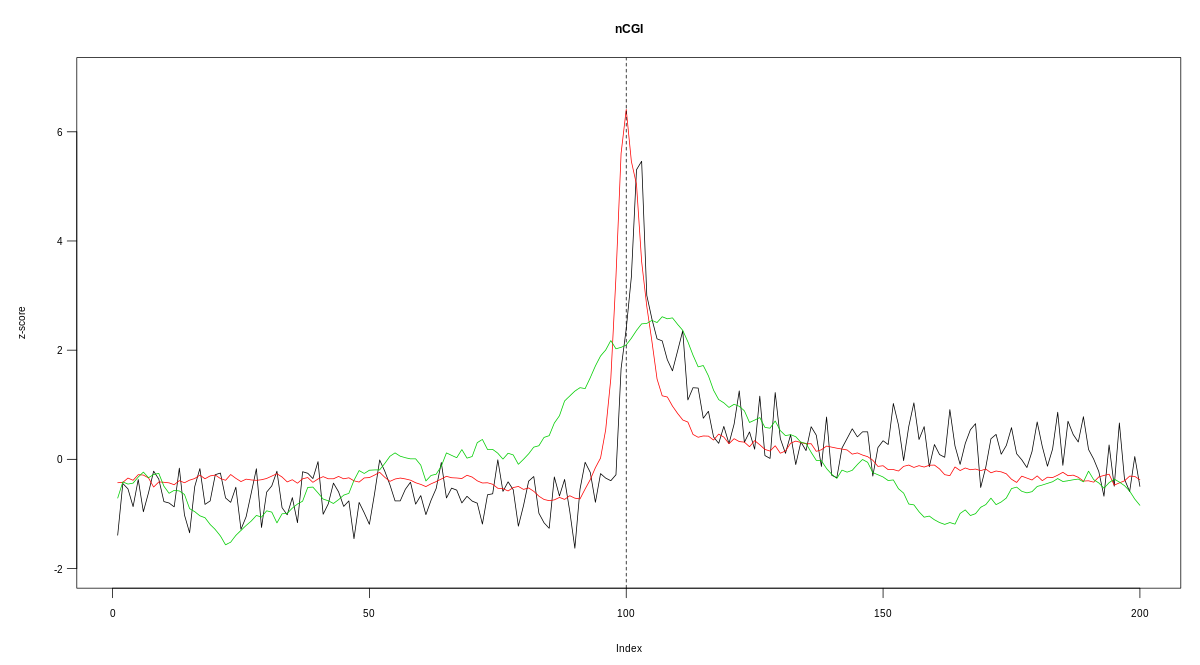

Supplement: Figure S4 — Overlapping normalized values of BGS, BBS and BVF-delta values for nCGI-TSSs. The z-scores for BBS (green line), BGS (red line) and BVF-delta (black line) are plotted for the same region. On the x-axis is the position of the bin relative to the TSS. (TIFF) [file pone.0114432.s004.tiff]

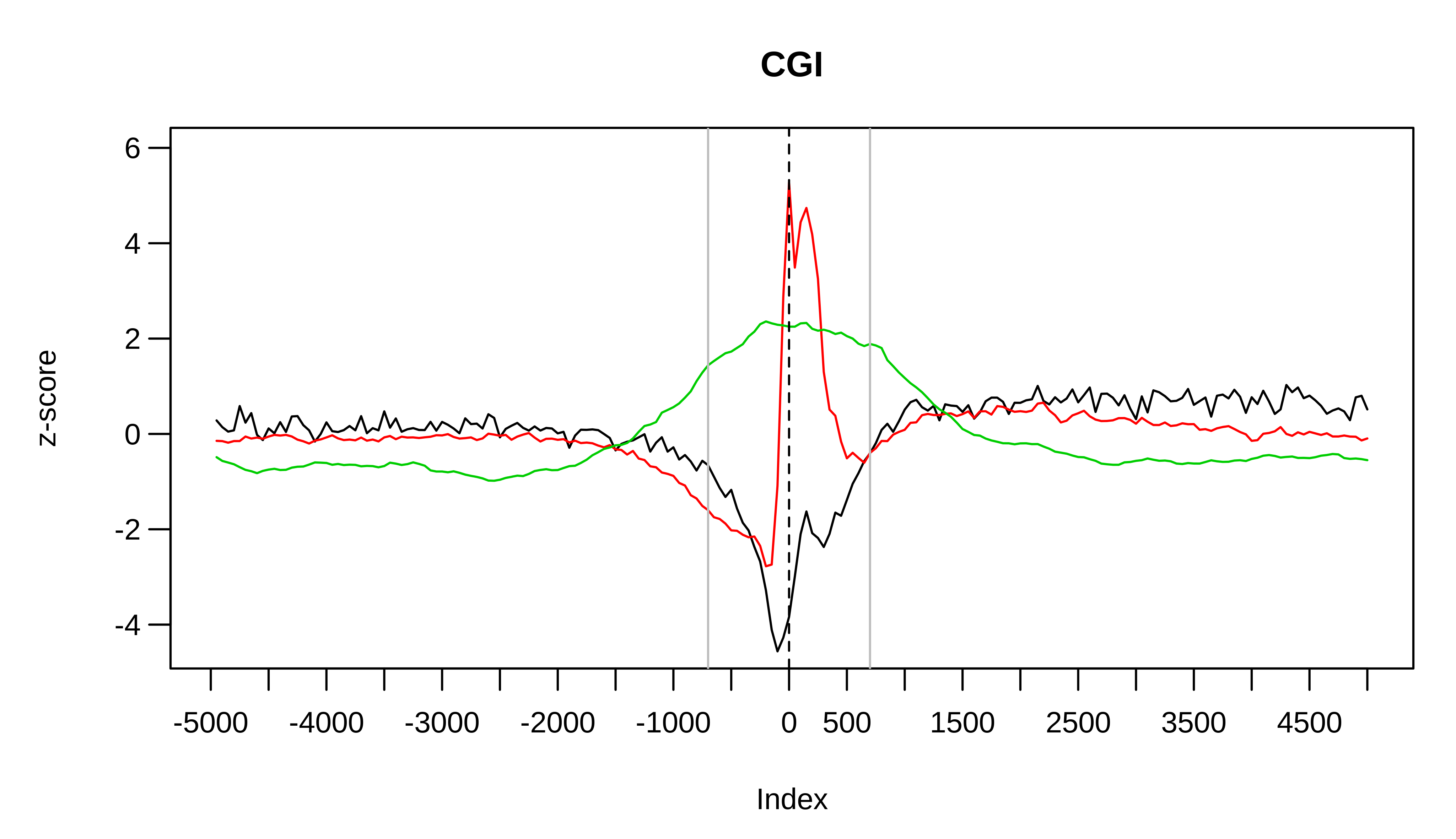

Supplement: Figure S5 — Overlapping normalized values of BGS, BBS and BVF-delta values for CGI-TSSs. The z-scores for BBS (green line), BGS (red line) and BVF-delta (black line) are plotted for the same region. The grey lines delimit the region defined under strong gBGC influence. On the x-axis is the position of the bin relative to the TSS. (TIFF) [file pone.0114432.s005.tiff]

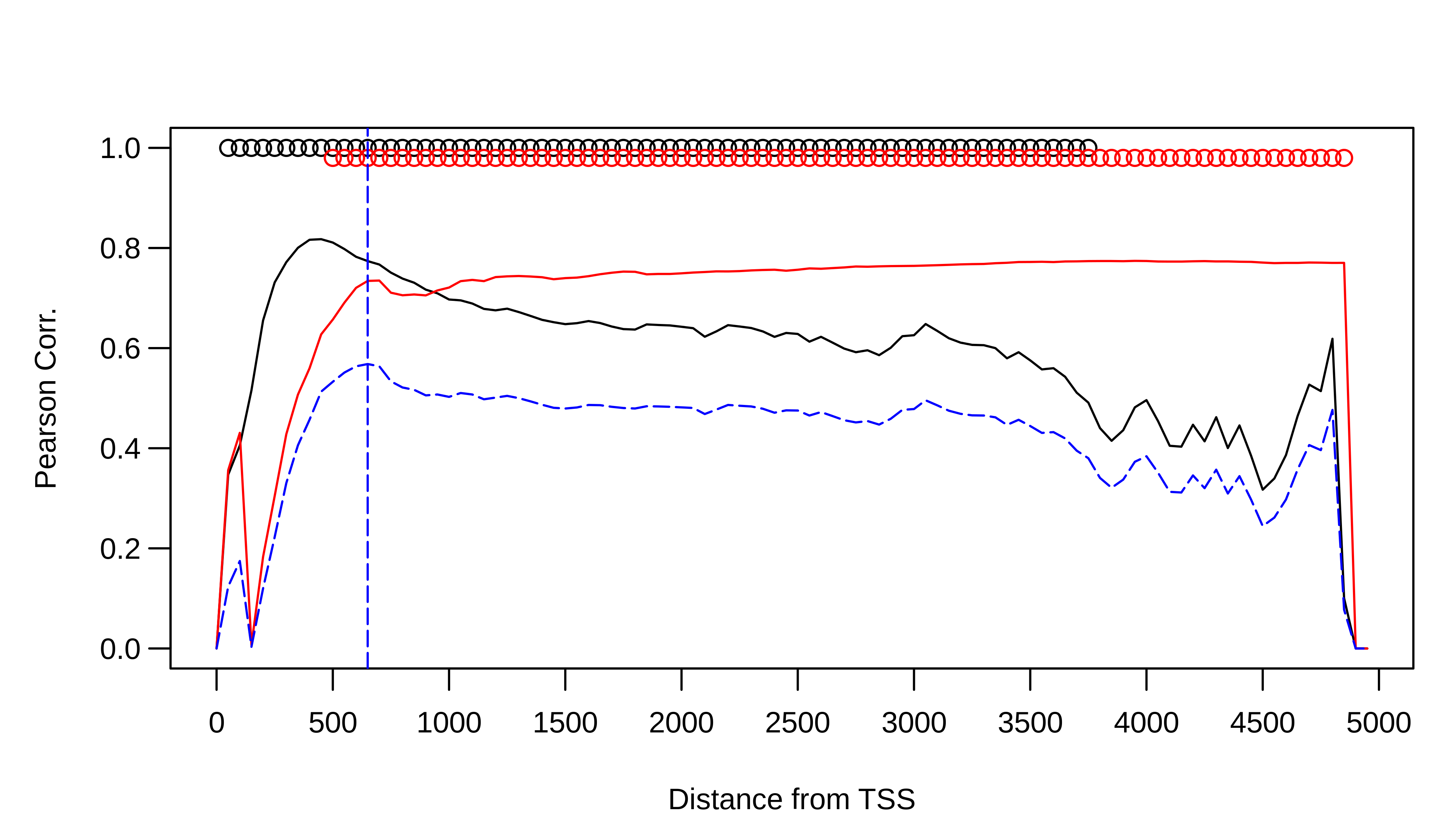

Supplement: Figure S6 — Regions under strong BBS and/or BGS influence. Figure shows, for each pair of inner-outer regions defined by the distance reported on the x-axis, the absolute value of the Pearson correlation for BBS and BVF-delta in the inner region (red line), the absolute value of correlation for BGS and BVF-delta in the outer region (black line) and the product of the two correlations (blue-dashed line). Red dots are placed where the correlation between BBS and BVF-delta is statistically significant and black dots are placed where the correlation between BGS and BVF-delta is statistically significant. The vertical dashed line represent the distance for which the value of the product of the two correlations is maximized. (TIFF) [file pone.0114432.s006.tiff]
