# Supplementary material for: Evidence for Evolutionary and Nonevolutionary Forces Shaping the Distribution of Human Genetic Variants near Transcription Start Sites
Source: PLoS One. 2014 Dec 4;9(12):e114432. doi: 10.1371/journal.pone.0114432 (PMC4256220; doi:10.1371/journal.pone.0114432)
Supplement: Data S1 — Instructions for obtaining data. (DOCX) [file pone.0114432.s007.docx]

Detailed instructions for obtaining data

**Genomic coordinates of human TSSs:**

Trough the UCSC “Table Browser” web interface (<http://genome.ucsc.edu/cgi-bin/hgTables>):

1. Select clade: *Mammal*
2. Select genome: *Human*
3. Select assembly: *Feb. 2009 (GRCh37/hg19)*
4. Select group: *Regulation*
5. Select track: *SwitchGear TSS*
6. Select table: *switchDbTss*
7. Select region: *genome*
8. Select: *get output*

Or

Trough the UCSC MySQL server:

1. Connect to the MySQL server using the command: “*mysql --user=genome --host=genome-mysql.cse.ucsc.edu –A*”
2. Execute the SQL query: “*select * from hg19.switchDbTss*”

**Human genomic variants:**

Trough the UCSC “Table Browser” web interface (<http://genome.ucsc.edu/cgi-bin/hgTables>) :

1. Select clade: *Mammal*
2. Select genome: *Human*
3. Select assembly: *Feb. 2009 (GRCh37/hg19)*
4. Select group: *Variation*
5. Select track: *All SNPs(138)*
6. Select table: *snp138*
7. Select region: *genome*
8. Select: *get output*

Or

Trough the UCSC MySQL server:

1. Connect to the MySQL server using the command: “*mysql --user=genome --host=genome-mysql.cse.ucsc.edu –A*”
2. Execute the SQL query: “*select * from hg19.snp138*”

**Genomic coordinates of CGIs:**

Trough the UCSC “Table Browser” web interface (<http://genome.ucsc.edu/cgi-bin/hgTables>) :

1. Select clade: *Mammal*
2. Select genome: *Human*
3. Select assembly: *Feb. 2009 (GRCh37/hg19)*
4. Select group: *Regulation*
5. Select track: *CpG Islands*
6. Select table: *cpgIslandExt*
7. Select region: *genome*
8. Select: *get output*

Or

Trough the UCSC MySQL server:

1. Connect to the MySQL server using the command: “mysql --user=genome --host=genome-mysql.cse.ucsc.edu –A”
2. Execute the SQL query: “select * from hg19.cpgIslandExt”

**Nucleosome localization data:**

1. Download the files
   1. <http://hgdownload.cse.ucsc.edu/gbdb/hg19/bbi/wgEncodeSydhNsomeGm12878Sig.bigWig>.
   2. <http://hgdownload.cse.ucsc.edu/gbdb/hg19/bbi/wgEncodeSydhNsomeK562Sig.bigWig>.
2. Then convert them to bedGraph format by using *bigWigToBedGraph* UCSC tool.

**GERP evolutionary scores:**

1. Download the file <http://hgdownload.cse.ucsc.edu/gbdb/hg19/bbi/All_hg19_RS.bw>.
2. Then convert it to bedGraph format by using *bigWigToBedGraph* UCSC tool.

**gBGC scores:**

1. Download the file <http://hgdownload.cse.ucsc.edu/gbdb/hg19/bbi/phastBiasPosteriors3.bw>.
2. Then convert it to bedGraph format by using *bigWigToBedGraph* UCSC tool.

**CADD scores:**

1. Download the file <http://krishna.gs.washington.edu/download/CADD/v1.0/1000G.tsv.gz>.
